# Imagination, Creativity, Napkins, and Persistence

**DOI:** 10.1016/j.jacadv.2022.100056

**Published:** 2022-08-26

**Authors:** David R. Holmes, Mohamad Alkhouli

**Affiliations:** Department of Cardiovascular Medicine, Mayo Clinic, Rochester, Minnesota, USA

**Keywords:** left atrial appendage, TAVI

“Without a whiteboard, without a sketchpad… in a moment of dilemma you have grabbed for the only thing you could find…a napkin”

Jon Hallsten^1^

The development and eventual dispersion of new technology is of fundamental importance as medicine approaches new clinical problems and solves current unmet patient and societal needs.

It is enhanced by new scientific data about adverse clinical outcomes of specific diseases as well as unanticipated problems which have been felt to be overcome. Multiple components, stages, and interactions are required. These begin with imagination (also known as the light switch turns on as per Edison) and then move often sequentially to creativity, napkins, and then the need for continued persistence. In all stages, serendipity often plays a significant role. These stages can be identified, and lessons were learned from 2 examples among many in our history of interventional cardiology. Dr Henning Andersen, then a research medical school student in Denmark, was given an unexpected opportunity by his department head to travel to a conference in Scottsdale, Arizona, in 1989. During a lecture given by Julio Palmaz, he saw examples and application of mechanical scaffolds (stents) to either improve flow or decrease and reverse complications of percutaneous transluminal coronary angioplasty (PTCA) in patients with coronary artery disease. During this lecture, like turning on a light bulb, Henning had the thought that a larger mechanical scaffold with folded valves within it could be developed and used to treat patients with severe aortic stenosis. Prior to that time, surgical aortic valve replacement was the only option for a progressive, ultimately fatal disease.

Analogous experiences like this have been described in interventional cardiology. The initial description of the field was described by Charles Dotter in 1963. At that time, he described the experience as, “The initial use of transluminal angioplasty was inadvertent. Early in 1963 a catheter was placed retrogradely through an occluded iliac artery. Although the chief accomplishment was diagnostic, the procedure led to the thought that one patient’s problem might be the route to another’s gain.” Subsequently, Andreas Grüntzig became inspired by Dotter’s experience to develop PTCA and performed the first in-man PTCA in 1977. Julio Palmaz was then inspired during a lecture given by Andreas about PTCA and, in a remembered history on a napkin, developed the first balloon expandable coronary stent. With that background burst of imagination and creativity, Henning then continued with the process.

During his flight home to Denmark, he formulated the 5 requirements for this method on a napkin: “1. Performed by retrograde catheterization; 2. A closed chest procedure; 3. A closed heart procedure; 4. A beating heart procedure; and 5. Performed without cardiopulmonary bypass.”^2^ With those napkin-documented considerations, he arrived home. After discussions with peers, supervisors, and others, it was felt that his idea would never work and be associated with fatal complications. In many ways, his experience followed the concepts elucidated by Thomas Kuhn^3^ in his definition of a paradigm shift using Albert Einstein’s words “if at first the idea is not absurd, then there is no hope for it.” Henning then further consulted with 1 professor who told him to “give it a try.”^2^

Following that, the theory of Calvin Coolidge and Hannaford came fully into being.^4^ “Nothing can take the place of persistence. Talent will not; nothing is more common than unsuccessful men with talent. Genius will not; unrewarded genius is almost a proverb. Education will not; the world is full of educated derelicts. Persistence and determination are omnipotent.” Armed with that strategy, Henning persisted. Within a few months, he took his invention from napkin drawings into functional prototypes, aided only by a surgical colleague in training, by self-paying for the needed basic materials that he purchased from a local hardware store. Henning articulated this breakthrough invention in a patent that will later be known as the “Andersen Patent” (Figure 1). The “Transcatheter Aortic Valve Implantation invention” was presented for the first time on May 19, 1990, at the 30-year anniversary symposium of the Danish Society of Cardiology in Odense, Denmark. This and another abstract formed the basis of a submission to the *Journal of the American College of Cardiology* in June 1990. Two of the 4 reviewers rejected it out of hand as “lacking long-term follow-up on durability, dislodgement, thrombus formation, inadequate improvement in hemodynamics”, among other concerns. It was eventually rejected with the all too familiar refrain “I am sorry to have to reject it, but the overall rating is that it has too low a priority for publication” (Figure 1A). It was also rejected by *Circulation* (Figure 1B) for which 1 of the reviewers stated, “I do not see any possible use of it in patients with calcified aortic stenosis.” Eventually, it was published in the *European Heart Journal*.^2^^,^^5^

**Figure 1:**
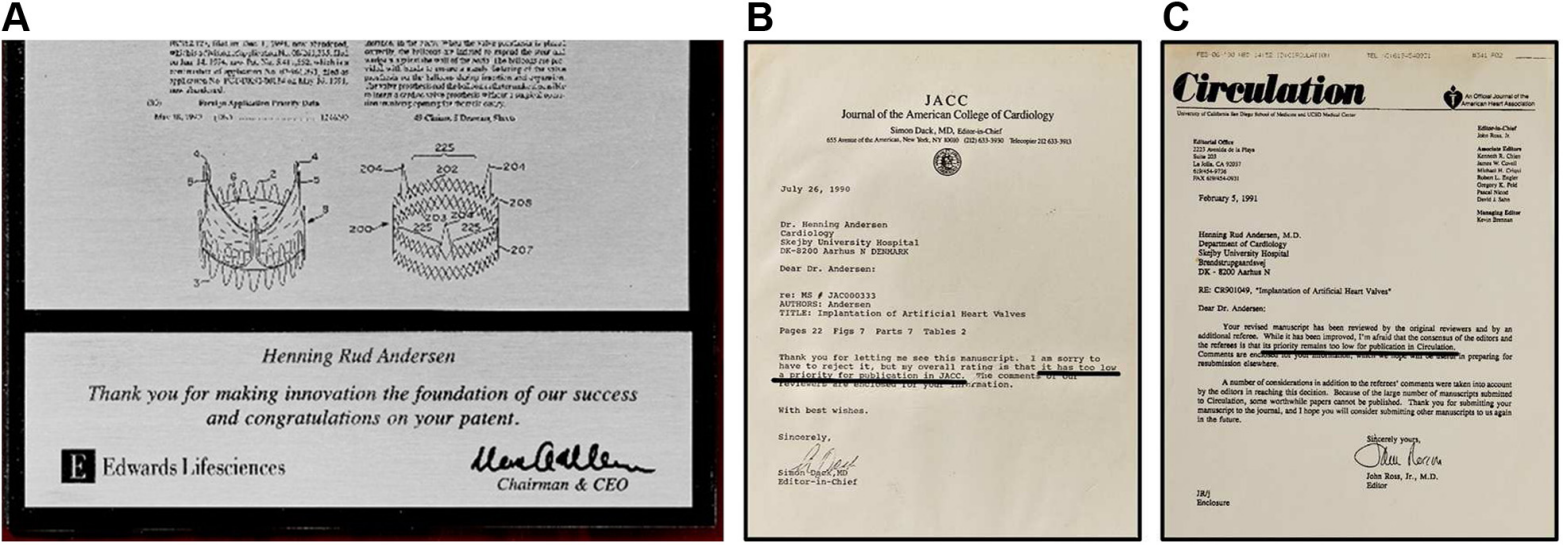
The Journey From Patent to Publication Is Not Always Easy **(A)** Excerpt of original Andersen Patent, November 17, 1989. **(B)***Journal of the American College of Cardiology* rejection of the transcatheter aortic valve implantation (TAVI) paper, 1990. **(C)***Circulation* rejection of TAVI paper, 1991. Images courtesy of Dr Henning R. Andersen. Figure reproduced with permission from Andersen. *Front Cardiovasc Med*. 2021:29;8:722693 (Creative Commons Attribution License—CC BY).

During and after this time, there were multiple other steps in the process of development that Henning describes as a “Doctor in Patent Courts”, with multiple interactions, endless negotiations, and experiments and discussions with several companies over intellectual property, some of which were extremely difficult and discouraging.^2^ Again, as per Calvin Coolidge, persistence, observations, belief, and determination were omnipotent in the process and fulfilled the fact that transcatheter aortic valve implantation has been a paradigm shift for the treatment of aortic valve stenosis and has inspired the development of new catheter treatments for structural heart disease.

The second example of this process from imagination and creativity, napkins, and persistence can be seen in the field of left atrial appendage (LAA) occlusion.^6^ This began in a hotel restaurant across from the Minneapolis-Saint Paul airport in 1997-1998. Three interventional cardiologists from 2 institutions—Drs David Holmes, Robert Schwartz, and Robert van Tassel—had been meeting regularly after hours to discuss new approaches to a variety of clinical issues and unmet clinical needs. Conversations included discussion of these needs and design of new strategies or approaches. There was great interest in the field of atrial fibrillation based on the clinical experiences each cardiologist had had with this condition in both clinical cardiology and electrophysiology. Discussion centered around an article that they had reviewed from a summary of data on the pathophysiology of stroke in that setting^7^ in which the finding was that strokes in patients with nonvalvular atrial fibrillation typically result from thrombus that originates in the LAA. After a series of discussions, that article stimulated the thought that perhaps by sealing the LAA off from the circulation, stroke could be prevented. Another moment of creativity and imagination when the light bulb was turned on and then a design of a potential looking device which was sketched out at that time on a napkin. This set the next stages of the development of this new technology for local site-specific treatment for stroke prevention.^6^

During these next stages, imagination and creativity were still needed as they worked together along with an expert patent attorney and Mayo Medical Ventures. Working with these stakeholders, a new start-up firm was identified (Atritech, Plymouth, Minnesota) which had been involved in technology development with Dr Van Tassel. Out of this came early prototypes with engineers specifically hired for this purpose. The 3 napkin people would meet on a regular basis to discuss issues, timelines, and review new designs. Throughout this time, other issues developed with another patent and a similar device design.^7^ After intense conversations, our current design was significantly modified to include a polytetrafluoroethylene coating for sealing the LAA, rather than a porous membrane that had been described with the other device.

From there, the project continued with the addition of engineers, consultants, animal experiment designs, and manufacturing considerations. The design of in vivo animal experiments was of crucial importance as it was an implantable device meant as an elective procedure in a field in which oral anticoagulants were the standard of care. There were considerations as to why such a device would be needed, how it would heal, would it be effective, as well as what periprocedural medications would be used. At last, by 2001, there was a product that could be deployed in patients. In consultation with the Food and Drug Administration (FDA), a pilot European first-in-human study was successfully completed resulting in excellent outcomes setting the stage for a first-in-human experience and then an initial randomized clinical trial in the United States.

Close discussions were held with statisticians, FDA, and other regulatory agencies.^8^ The specific endpoints of the trial were carefully considered in terms of primary and secondary endpoints. This process began to require the concept of persistence. After that first trial, Atritech and the investigators presented the results to the FDA panel. The conclusions of the panel meetings have been well documented. In summary, it required 3 panel meetings using the same protocol with the same device for the same indication to finally receive FDA approval in 2016 after the first use in the United States in patients had occurred in 2002.^9^ During this time, the initial company, sales forces, and intellectual property was purchased by the Boston Scientific Corporation. That initial device, after the 3 panel attempts as a first-in-kind class, has now been tested and used in more than 500,000 patients worldwide and spawned a whole category of interventional devices.^10^

In a field such as interventional cardiology, new devices, approaches, and technology are needed as unmet clinical needs, and new and expanded groups of patients are identified. These 2 examples document approaches that have been taken and lessons learned. Clinical information about patients and diseases is of fundamental importance on which imagination is overlaid which has been described as the only thing which limits our ability. If the device appears promising, patents should be strongly considered either within the institution of the authors or, if not available, with outside counsel. An extremely important item relates to the fact that the potential device should never be disclosed publicly either in print or at any public meeting or with other people who may have conflicts of interest until the patent application process has been completed. Failure to follow that may result in loss of any patent protection. Following those initial steps, the processes taken in these 2 examples are of great importance and may include, among other things, basic laboratory testing and eventually animal studies. Finally, as the device has moved along toward the design of early feasibility studies, discussion with the regulatory agencies in the country of origin are essential in terms of trial design, endpoints, and metrics of success.

Interventional cardiology is a field of expanding boundaries of patient care. Attention to the concepts of imagination, creativity, napkins, and persistence are essential.

## Funding support and author disclosures

Dr Alkhouli serves on the CHAMPION trial steering committee and advisory boards of Boston Scientific and Philips. Dr Holmes has reported that he has no relationships relevant to the contents of this paper to disclose.
